# Targeting therapy for homocysteic acid in the blood represents a potential recovery treatment for cognition in Alzheimer's disease patients

**DOI:** 10.18632/aging.101046

**Published:** 2016-09-14

**Authors:** Tohru Hasegawa, Wataru Ukai

**Affiliations:** ^1^ Saga Woman Junior College, Saga 840-8550, Japan; ^2^ Department of Neuropsychiatry, Sapporo Medical University, School of Medicine, S-1, W-16, Chuo-ku, Sapporo, 0608543, Japan

**Keywords:** Alzheimer's cognitive decline, homocysteic acid in peripheral blood, homocysteic acid in urine, decreasing homocysteic acid in blood

## Abstract

At present, we have no reliable means of recovering cognitive impairment in Alzheimer's disease (AD) patients. We hypothesized that homocysteic acid (HA) in the blood might represent one such pathogen that could be excreted into the urine. Since DHA is known to reduce circulating levels of homocysteine, and since exercise attenuates this effect, it follows that supplementation of the diet with DHA, along with increased levels of physical activity, may help to reduce cognitive impairment in AD patients. Our hypothesis was proven to be correct because memory problems in 3xTg- AD mice (a model for AD in which animals develop amyloid pathology), and in a mouse model of familial AD, were recovered following treatment with an anti-HA antibody and not by amyloid treatment. Interestingly, 3xTg-AD mice with amyloid pathology showed increased levels of HA level. This could perhaps be explained by the fact that amyloid precursor protein and/or presenilin increases calcium influx, which could then increase levels of superoxide and consequently increase levels of HA from homocysteine or methionine. Our hypothesis is also partially supported by an open clinical trial of certain dietary supplements that has shown impressive results. Also there are other treatments hypothesis which would be possible for the effective therapies, such as ribonucleoprotein therapy, a β-secretase inhibitor treatment and the metabolic enhancement treatment.

Amyloid cascade hypothesis is strong and main stream hypothesis in Alzheimer's field [1]. The enormous articles of amyloid toxicities were published and the antibody which reduces a quantity of amyloid has been developed and finally this therapy was conducted for human Alzheimer.

Recent big two clinical trials of amyloid beta treatment for Alzheimer's disease were all failed to recover the cognitive impairment [2, 3], it has forced us to reconsider this central hypothesis of amyloid pathogen for Alzheimer's disease. Yes, it is very obvious that AD patients have a large amount of amyloid beta in their brains [4]. However it is also obvious that normal brain also accumulates amyloid beta, and this normal brain can show the normal cognitive ability [5]. This is a key point. The amyloid cannot disturb the normal brain work. But why did many papers report its toxicity? And why did the clinical trial of amyloid treatment not succeed to recover the cognition? First we should consider that many amyloid toxicities were observed in animal experiment, especially the amyloid gene activated model mice were used. It is a famous scientific fact that human cannot synthesize vitamin C, but mice can synthesize vitamin C [6]. And human case of Alzheimer's disease, amyloid pathology is usually observed with aging, but mice do not accumulate amyloid with aging. Yes, it is obvious that mice has more powerful anti-oxidant efficacy than human and we should keep a mind that mice model for AD are carefully considered to adapt for human case.

Second if the amyloid beta cannot induce a toxic cascade flow by an unknown physiological effect, amyloid pathology will not show any degenerative effect to the neuron and consequently amyloid accumulation will occur in the normal brain. That is a next point. It is known that the amyloid pathology stimulates the calcium influx [7], which stimulate oxygen radical formation [8] to produce homocysteic acid from homocysteine or methionine. And if some physiological antioxidant effect will be large enough, this oxygen radical formation will not be produced and consequently will not produce such a toxic HA. Now we hypothesize why normal brain can accumulate the amyloid beta [5] and that the cognitive reserve ability will be shown in such a typical AD brain.

The familial AD [9] will be observed by a such amyloid mechanism, because it is well known that some genes such as APP and/or Presenilina induces a familial AD and these genes stimulate calcium influx [7, 10]. The stimulated calcium induces the superoxide formation [8, 45] to produce HA from homocysteine or methionine. However if the familial AD patient's brain can show the effective antioxidant power to the amyloid pathology, the amyloid toxicity does not appear until their physiological antioxidant power will decrease. The produced HA can destruct the cognitive reserve ability and consequently induces AD pathology. The familial AD (FAD) pathology will be occurred in an earlier age. Because FAD genes will be activated in an earlier age. The amyloid pathology will not be occurred in some physiological conditions.

Also some papers reported the physiological effect of amyloid on neurons [11–34]. For example, low concentration (picomole) of amyloid could act as trophic factor [11–15] and APP (amyloid precursor protein) could act as neuronal controller [13, 17]. Also low concentration of amyloid beta could act as neurological modulator [18–24]. And anti-oxidant effect of amyloid is very interesting [23–25], because it is well-known that aging process induced oxidative stress and the aging brain contained more amount of amyloid that younger one.

Recently some papers have reported that our peripheral blood may have some pathogens for the AD cognitive impairment, not in the brain itself.

In earlier studies published in Nature [35, 36], scientists at the Stanford University School of Medicine identified substances in the blood of old mice that made the brains of younger mice act older. These substances, whose levels rise with increasing age, appeared to inhibit the brain's ability to produce new nerve cells critical for memory and learning. These findings raise the question of whether it is possible to shield the brain from aging by eliminating or mitigating the effects of these apparently detrimental blood-borne substances.

APOE4 is a gene involved in the mechanisms underlying the development of AD. It increases permeability of the blood–brain barrier (BBB) [37], which thus allows pathogens present in the peripheral blood to readily traverse the BBB and ultimately disturb brain function. This report indicates the possibility that APOE4 represents an early-onset gene.

Another report [38] described an early AD patient showing destruction of the hippocampal BBB. However, this report did not describe which blood-borne pathogen was involved or the causative factor underlying increased permeability of the BBB in aged hippocampus. It is possible that these same blood-borne pathogens can be excreted in the urine as a result of increased blood circulation during exercise, which would thus stop the cognitive decline of AD patients. It also follows that the pathogens involved cannot be large molecules such as proteins, as these cannot be excreted into the urine. As yet, the precise identity of the small molecule pathogens involved remains elusive.

It has been reported that HA is a probable blood-borne pathogen for AD [39–42] and increases BBB permeability via the activation of HA by NMDA receptors [43]. HA is a known glutamate receptor agonist [44] with a molecular weight of 183 KDa. JAD [39] study reported a significant negative relationship between MMSE scores and HA levels in the blood. Consequently, it follows that HA might represent one of the blood-borne pathogens responsible for AD.

We already observed the positive statistical significant relationship between an urinary HA level and the MMSE score, which indicated that the cognitive function was high if there were many urinary HA discharges [39]. However, a key point to consider is how HA might be related to the amyloid pathology of AD. First, it is important to consider the potential neurodegenerative effects of HA. An earlier study [41] described such degenerative effects of HA. The mouse model for AD, the 3xTg-AD mouse, was originally developed by Professor Laferal and was created specifically to develop amyloid pathology that destroyed normal memory function. It has been shown that memory problems in this mouse model can be rescued by treatment with an anti-HA antibody. It should be pointed out that this was a polyclonal anti-HA antibody, which could have reacted with other HA-related compounds, although our own observations confirmed that HA levels in the brain were indeed reduced following treatment with this antibody. It is also worth noting that HA is completely independent of amyloid toxicity. Since 3xTg-AD mice are a good model for familial AD and that an anti-HA antibody can rescue memory problems in this mouse model, it follows that an anti-HA antibody could potentially recover amyloid-induced cognitive impairment in familial and other types of AD in humans.

However, the question remains as to why 3xTg-AD mice exhibited increased HA levels that were not related to amyloid pathology. Recent studies have shown that APP and Presenilin stimulate calcium influx [8, 10], which then stimulates the production of superoxide radicals [8], and these superoxide radicals stimulate the production of HA from methionine and homocysteine [45]. Collectively, these reports suggest that HA levels increase via amyloid pathology. However, this does not explain why amyloid treatment is unable to rescue cognitive impairment. This suggests significant differences between the relevant physiology of mice and humans. In humans, there is increased production of HA in the brain from amyloid, and increased levels of HA in the blood, with the progression of age [39]. However, HA levels are not known to increase with aging in mice (unpublished observation). Consequently, amyloid treatment can recover cognitive impairment in mice. This aging effect in humans can still destroy cognitive function following amyloid treatment. A recently published article reported that the amino acid, methionine, can induce the formation of amyloid plaques, characteristic pathological changes associated with AD, via hosphorylated-tau protein [46]. This is particularly interesting because HA can be produced from heavily-oxidized methionine [8, 45]. Therefore, we can hypothesize that amyloid treatment, in combination with other treatments such as anti-HA treatment, are likely to show positive curative effects.

Presently, a range of treatments are under evaluation such as those that prevent inflammation or the phosphorylation of tau or involve mitochondrial treatment [47]. And the metabolic enhancement treatment [48], β-secretase inhibitor treatment [49] and ribonucleoprotein therapy [50] are also interesting hypotheses. Such treatments appear to be effective and are very promising. However, it is important to consider that these treatments may involve drugs that may have deleterious side effects.

The development of an effective anti-HA therapy requires consideration of several factors. Firstly we need to take into account the fact that HA in the blood will be discharged into the urine by stimulating the circulation of blood. Aerobic exercise stimulates the blood circulation to stimulate the discharge of HA discharge. A recent paper showed that aerobic exercise was very effective in preventing AD pathology [51].

We conducted the open clinical trial of HBF supplement for the cognitive recovery of 91 AD patients of all stage. This HBF supplement decreased the HA levels in blood, which induced the remarkable cognitive recovery processes of all stages of AD patients (unpublished data). From our open clinical trial, our hypothesis is proved. Targeting treatment of HA in blood will be useful and effective therapy for the cognitive recovery of AD patients. Our clinical trial presented the one powerful answer to the question. That is, when the amyloid treatment failed to recover the cognition, it is thought that the treatment time was too late to recover the patients's cognition, because the patients' neurons were already lost. However our trial presents that patient's cognition could be recovered as such. Of course, the final stage of patient could not recover his cognition as normal stage, because such as hippocampus organ already degenerated. The regenerative medicine becomes important at this stage. However we should keep a mind that the regenerative therapy should be conducted after the pathogen such as HA will be treated.

Our hypothesis, the destruction of HA will induce the cognitive reserve ability and its realization is urgent hypothesis to be solved.
